# Network Pharmacology and Molecular Modeling Techniques in Unraveling the Underlying Mechanism of Citri Reticulatae Pericarpium aganist Type 2 Diabetic Osteoporosis

**DOI:** 10.3390/nu16020220

**Published:** 2024-01-10

**Authors:** Jiangtao Li, Ying Wang, Amin Ullah, Ruiyang Zhang, Yuge Sun, Jinjie Li, Guangning Kou

**Affiliations:** 1 Centre for Nutritional Ecology and Centre for Sport Nutrition and Health, Zhengzhou University, Zhengzhou 450001, China; 2 Department of Nutrition and Food Hygiene, School of Public Health, Zhengzhou University, Zhengzhou 450001, China

**Keywords:** Citri Reticulatae Pericarpium, type 2 diabetic osteoporosis, network pharmacology, molecular docking, molecular dynamics simulations

## Abstract

Type 2 diabetic osteoporosis (T2DOP) is a common complication in diabetic patients that seriously affects their health and quality of life. The pathogenesis of T2DOP is complex, and there are no targeted governance means in modern medicine. Citri Reticulatae Pericarpium (CRP) is a traditional Chinese medicine that has a long history and has been used in the treatment of osteoporosis diseases. However, the molecular mechanism for the CRP treatment of T2DOP is not clear. Therefore, this study aimed to explore the underlying mechanisms of CRP for the treatment of T2DOP by using network pharmacology and molecular modeling techniques. By retrieving multiple databases, we obtained 5 bioactive compounds and 63 common targets of bioactive compounds with T2DOP, and identified AKT 1, TP 53, JUN, BCL 2, MAPK 1, NFKB 1, and ESR 1 as the core targets of their PPI network. Enrichment analysis revealed that these targets were mainly enriched in the estrogen signaling pathway, TNF signaling pathway, and AGE-RAGE signaling pathway in diabetics, which were mainly related to oxidative stress and hormonal regulation. Molecular docking and molecular dynamics simulations have shown the excellent binding effect of the bioactive compounds of CRP and the core targets. These findings reveal that CRP may ameliorate T2DOP through multiple multicomponent and multitarget pathways.

## 1. Introduction

Diabetes of type 2 is a type of diabetes that is more prevalent in young and older adults. Those with type 2 diabetes comprise 90% of the total number of diabetic patients in China [1]. Diabetic osteoporosis, a prevalent complication of Type 2 diabetes, is characterized by pain and fractures that result from a disruption in bone metabolism and a degradation of bone microstructure [2]. Studies have indicated that individuals diagnosed with type 2 diabetes are at an increased risk of developing osteoporosis. At the same time, compared with general age-related osteoporosis, patients with type 2 diabetes osteoporosis tend to be more susceptible to bone fragility due to prolonged exposure to a high-glucose environment [3]. Hence, as the global population ages, the severity of the situations regarding type 2 diabetic osteoporosis will also increase [4].

Modern medical treatment for Type 2 diabetic osteoporosis (T2DOP) usually consists of managing the underlying condition and supplementing with anti-osteoporosis medications and calcium supplements; however, the efficacy of these approaches is not readily evident. Additionally, the use of these anti-osteoporosis medications could cause adverse reactions as a result of dose dependence and bodily resistance [5]. Finding a novel therapeutic intervention for T2DOP is, thus, an imperative concern. Traditional Chinese medicine (TCM) is well known and holds a distinctive position in China. TCM has long been used as a prevalent therapeutic approach in China and throughout Asia. The value of TCM is becoming increasingly acknowledged in modern society, and its beneficial medicinal properties and low cost make it more suitable for patients. In contrast, recent disease therapies frequently incorporate TCM into treatments due to its multifaceted composition and complex mechanism of action [6].

Citri Reticulatae Pericarpium (CRP) is one of the most widely used herbs in TCM. It was first included in the Herbal Classic of Materia Medica by Shennong. It is renowned as “the king of mediating drugs” and is the origin of food and medicine homology. Produced mainly in southern China, CRP has the effects of qi, moisture, and phlegm [7,8]. Research has shown that CRP is rich in natural active substances such as nobiletin, naringenin, hesperidin, etc., which have good anti-inflammatory, antioxidant, and anticancer functions. Modern medicine increasingly utilizes CRP to treat metabolic diseases due to its beneficial medicinal properties [9,10,11]. According to traditional Chinese medicine, osteoporosis is caused by the insufficiency of vital energy and blood [12]. CRP, the prominent qi medicine, has great potential in the treatment of osteoporosis. Recent scientific investigations have further verified the protective effects of CRP and its bioactive components on bones [13,14,15,16,17]. Due to the complexity of CRP’s components, however, the precise targets and pathways by which CRP ameliorates T2DOP remain unknown; further studies are needed to investigate these targets and pathways.

Due to the continuous advancements in numerous drug and disease databases, the utilization of bioinformatics and network pharmacology techniques to identify novel drugs and investigate disease treatment mechanisms has gained significant attraction in recent years. The drug–component–target–disease network it offers has become a significant means of traditional Chinese medicine research [18]. Therefore, this study aims to investigate the molecular mechanisms and primary targets of CRP and its bioactive components in an effort to ameliorate osteoporosis associated with type 2 diabetes using network pharmacology technology. To further validate the binding capability of the bioactive components and the main targets, molecular docking and molecular dynamics simulation methods were implemented.

## 2. Materials and Methods

### 2.1. Bioactive Compounds of Citri Reticulatae Pericarpium (CRP) and Screening of Effect Targets

Effect targets and bioactive compounds were obtained by utilizing the Traditional Chinese Medicine Systems Pharmacology (TCMSP, https://tcmspw.com/, accessed on 15 October 2023) and BATMAN-TCM databases (http://bionet.ncpsb.org/batman-tcm/, accessed on 16 October 2023). We screened the active ingredients in the TCMSP database using “Citri Reticulatae Pericarpium” as the key term; OB criteria were values equal to or greater than 30% [19], and DL criteria were values equal to or greater than 0.18 [20]. Concurrently, the effect targets relating to these bioactive compounds were retrieved from the databases, as mentioned earlier. The BATMAN-TCM database is widely used to evaluate the effect targets of bioactive compounds in traditional Chinese medicine (TCM). The sources of these effect targets mainly come from two aspects, one from articles and experiments and the other from the prediction of the effect targets of related components. We established targets in the BATMAN-TCM database with druggable scores ≥0.1 and confidence scores ≥0.95. Only identified targets that served as complements to effective chemical composition targets were included.

### 2.2. Collection of Targets Related to Type 2 Diabetic Osteoporosis

By using “type 2 diabetic osteoporosis” as the search term, the relevant T2DOP targets were identified in the Genecards (www.genecards.org, accessed on 16 October 2023) and OMIM (https://www.omim.org/, accessed on 16 October 2023) databases, respectively. For the acquisition of targets in the two databases, we selected targets with scores greater than 25 and approved symbols as representative targets for T2DOP. Meanwhile, the acquired disease targets were examined using the UniProt database (https://www.uniprot.org/, accessed on 19 October 2023). Finally, these targets were merged, and the duplicates were removed.

### 2.3. Determination of the Common Targets of Citri Reticulatae Pericarpium and T2DOP

The bioactive compound targets of CRP and T2DOP related to these targets were entered into the online platform Interactivenn (http://www.interactivenn.net/, accessed on 22 October 2023) in order to derive the common targets of CRP and T2DOP and save the Venn diagram.

### 2.4. Protein–Protein Interaction Network Construction and Core Target Acquisition

For protein–protein interaction (PPI) network construction, the obtained common targets of CRP and T2DOP were entered into the STRING online data analysis platform (https://cn.string-db.org/, accessed on 22 October 2023) with the following biological attributes configured: “human,” “high confidence,” and “hidden free targets;” the remaining values were left as platform defaults. The PPI protein interaction data that were analyzed were imported into Cytoscape3.9.1 as a TSV file and utilized for visualization purposes. This software provided a graphical representation of the interactions between T2DOP and common target proteins of CRP. We used three screening techniques in order to identify the main targets of this protein interaction network with precision [21]. First, the top ten targets ranked by the number of nodes were included in the screening based on the analysis results of STRING. Second, the significance module analysis was performed using the Cytoscape plug-in tool MCODE. Third, using the cytoHubba plugin, the top ten hub genes were calculated in accordance with MCC. Finally, the final core targets were determined by comparing these genes.

### 2.5. GO and KEGG Pathway Enrichment Analysis

Metascape is the main database for bioprocess annotation and pathway enrichment; it comprises over 40 databases of biological information and is subject to regular updates. The common targets were entered into the Metascape platform (https://metascape.org, accessed on 23 October 2023) for Kyoto Encyclopedia of Genes and Genomes (KEGG) pathway enrichment analysis and gene ontology (GO) bioprocess enrichment analysis [22,23], selecting the “human” species, ensuring a minimum overlap of 3, setting the *p*-value at <0.01, and setting the enrichment factor at >1.5. The output files were arranged according to the *p*-value, and the top ten results were selected according to the molecular function (MF), biological process (BP), and cellular components (CC) as the main biological functions. Likewise, the primary pathway was determined from the top 20 pathways based on the size of the *p*-value. The analyses of these two components revealed the biological mechanisms and potential pathways that the CRP could potentially use to ameliorate T2DOP. Using the R programming language, the KEGG pathway enrichment analysis and GO analysis were subsequently visualized.

### 2.6. Construction of Target–Pathway Interaction Networks

Cytoscape 3.9.1 software was used to import the top 20 pathways, top 20 pathway-related targets, and bioactive compounds of CRP from the KEGG pathway enrichment analysis results. The software then extracted the correspondence between bioactive compounds, targets, and pathways, determined the target–pathway interaction relationship of the top 20 signaling pathways, and analyzed the pooling of target proteins.

### 2.7. Molecular Docking

The bioactive compounds of CRP and core target proteins were docked using Autodock Vina software, and the binding situation and binding energy size were analyzed. Initially, the SDF file containing the bioactive compounds was obtained from the PubChem database (https://pubmed.ncbi.nlm.nih.gov/, accessed on 28 October 2023). The file was then inputted into Chem3D software 20.0, where the MM2 function was applied to energy-minimize the structure. The resulting 3D structure was exported in mol2 format. The structures of the core targets were identified through a screening process utilizing the UniProt database. The screening criteria included validated, X-crystal diffraction-resolved protein structures in the human species that were less than 3.0A in resolution and had previously been used in the literature for molecular docking. The original structures of these targets were obtained from the PDB database and underwent ligand modification using Pymol 2.1 software. Subsequently, the modified core target and bioactive compounds were introduced into the Autodock Tools 1.5.7 for docking. Then, we analyzed the binding energy size and derived the conformation with the highest affinity.

### 2.8. Molecular Dynamics Simulation

Molecular dynamics simulation is a highly efficient technique for investigating the dynamic characteristics of small molecules and proteins. An Amber18 was used for molecular dynamics simulation in this study [24]. Hartree–Fock (HF)SCF/6-31G* calculation of the antechamber module and Gaussian 09 software were utilized to obtain the small molecule RESP charge utilized in the simulation [25]. Hydrogen atoms were subsequently incorporated into the protein–small molecule complex system described in the LEaP module for the GAFF2 small molecule and ff14SB protein force fields [26,27]. On the basis of small-molecule and protein complexes, an octahedral TIP3P solvent box was constructed with a 10 Å cutoff value [28]. To achieve charge balance in the system, Na^+^/Cl^−^ was introduced. Subsequently, the simulations generated the topology and parameter files.

We follow four phases throughout the simulation: energy minimization, heating, equilibrium, and finished product simulation. In the first phase, the energy optimization procedure involved conducting the steepest descent method with 2500 steps, followed by the energy minimization method using the conjugate gradient method with 2500 steps. The system was subsequently heated for 200 ps at a constant volume and heating rate during the heating phase, resulting in a gradual increase in temperature from 0 K to 298.15 K. The equilibrium was subsequently divided into two steps. First, 500 ps of NVT (isothermal body) ensemble simulation was performed at a system maintenance temperature of 298.15 K to further distribute the solvent molecules throughout the solvent box in an even pattern. A balanced treatment of 500 ps was performed in the second step using the NPT (isothermal and isobaric) ensemble simulation. The system pressure during the final product simulation phase was 1 atm, the integration step lasted 2 fs, and the trajectory for the 100 ns NPT (isothermal isobaric) ensemble simulation was saved every 10 ps. The simulation used the particle mesh Ewald (PME) method to calculate long-range electrostatic interactions [29], the SHAKE method to restrict the distance to hydrogen bonds [30], and the Langevin algorithm to control the temperature [31]. The cut-off distance for non-bonds was set to 10 Å.

### 2.9. Binding Free Energy Calculations of MMGBSA

The MM/GBSA method was calculated using the MMPBSA.py module based on the simulated traces [32,33,34,35]. In this study, the MD trajectories of 90–100 ns were sampled by the following formula:

$${\Delta G}_{\mathrm{bind}}={\Delta G}_{\mathrm{complex}}-({\Delta G}_{\mathrm{receptor}}+{\Delta G}_{\mathrm{ligand}})\;={\Delta E}_{\mathrm{internal}}+{\Delta E}_{\mathrm{VDW}}+{\Delta E}_{\mathrm{elec}}{+\Delta G}_{\mathrm{GB}}+{\Delta G}_{\mathrm{SA}}$$

where “ΔE_internal_” denotes the internal energy, “ΔE_VDW_” represents the van der Waals interaction, and “ΔE_elec_” represents electrostatic interactions, respectively. The internal energy consists of bond energy (E_bond_), angular energy (E_angle_), and torsion energy (E_torsion_); it also includes solvation-free energy, and G_GB_ and G_SA_ represent polar and nonpolar solvation-free energies, respectively. For ΔG_GB_, the GB model developed by Nguyen et al. [36] was used. The formula for calculating the nonpolar solvation-free energy (Δ G_SA_), which is the product of solvent accessibility surface area (surface area, SA) and surface tension (γ), was as follows: G_SA_ = 0.0072 × SASA [37].

## 3. Results

### 3.1. The Bioactive Compounds of Citri Reticulatae Pericarpium (CRP) and Effect Target Results

A total of 64 effect targets and five bioactive compounds were identified through screening in the TCMSP database. We conducted a search in the BATMAN-TCM database for these five bioactive compounds and discovered that 58 targets had been identified as having a confirmed link with them. By integrating the outcomes of these two database searches, we acquired 83 effect targets and five bioactive compounds in total (Table 1).

### 3.2. Targets Related to Type 2 Diabetic Osteoporosis (T2DOP)

The Genecards and OMIM databases were searched for 1470 and 184 eligible screenings for T2DOP-related targets. Following the elimination of duplicates, a total of 1612 targets were finally obtained for T2DOP.

### 3.3. Common Targets of Citri Reticulatae Pericarpium and Type 2 Diabetic Osteoporosis

**Figure 1:**
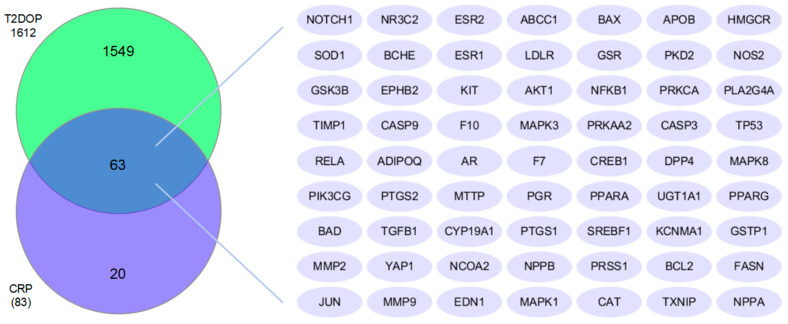
Common targets of both CRP and T2DOP.

The common targets of CRP and T2DOP numbered 63, as shown in the Venn diagram (Figure 1). We believe that these 63 common targets may be potential targets to be targeted by CRP in order to ameliorate T2DOP.

### 3.4. PPI Network and Core Target Analysis

**Figure 2:**
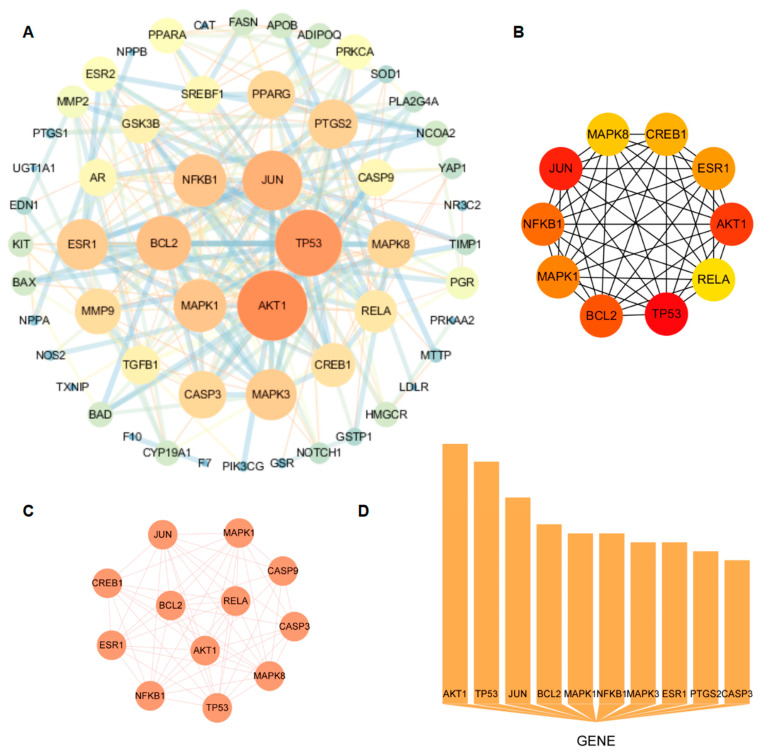
Protein–protein interaction network and results of three methods through which to calculate its core targets. (**A**) CRP and T2DOP common-target PPI network; (**B**) core targets screened by cytoHubba; (**C**) core targets screened by MCODE; (**D**) top 10 core targets in terms of node count.

A total of 63 nodes and 242 edges were identified through the analysis of 63 common CRP-T2DOP targets that were imported into the STRING database. As shown in Figure 2A, the protein interaction network diagram was generated by inputting the TSV file containing the analyzed results into the Cytoscape software. The size of the circle represents the degree value of the target, while the strength of the interaction between the two targets is denoted by the thickness of the line connecting them. The core targets in this network were AKT1, TP53, JUN, BCL2, MAPK1, NFKB1, and ESR1, which were determined by the intersection of the calculated results of the “cytoHubba” (Figure 2B) and “MCODE” (Figure 2C) plugins in the Cytoscape software and the top 10 in node counts (Figure 2D).

### 3.5. Common-Target GO Analysis and KEGG Pathway Analysis

**Figure 3:**
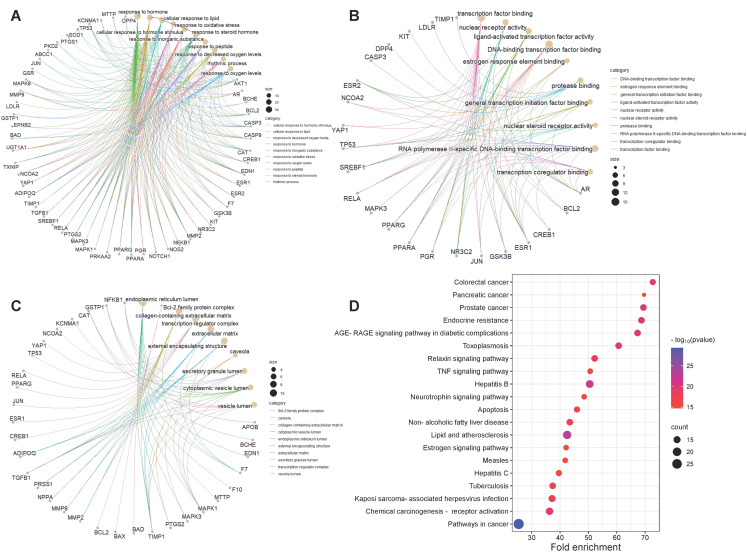
GO and KEGG enrichment analysis. (**A**) Biological process; (**B**) molecular function; (**C**) cell component; (**D**) top twenty signaling pathways.

**Figure 4:**
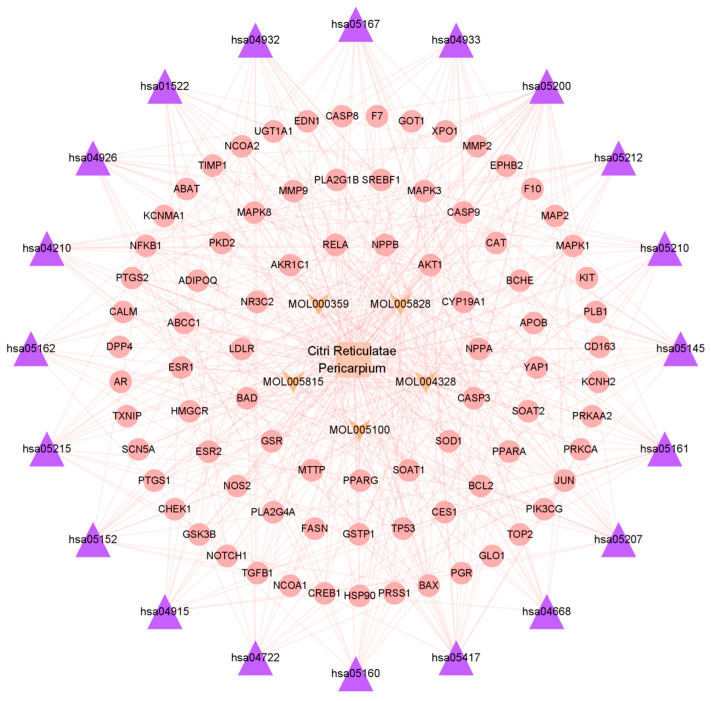
Bioactive compound–target–pathway network of Citri Reticulatae Pericarpium.

In order to obtain a more comprehensive understanding of how CRP and its bioactive compounds ameliorate T2DOP, 63 common targets of CRP-T2DOP were subjected to GO functional enrichment analysis and KEGG pathway enrichment analysis. The enrichment results showed 1228 GO entries (*p* ≤ 0.01), including 1108 BP, 76 MF, and 44 CC. According to the *p*-value size, the top 10 entries of each component were selected. The analysis revealed that the 63 common targets were primarily enriched in response to hormones and oxidative stress in biological processes (Figure 3A); in transcription factor binding and protease binding in molecular function (Figure 3B); and in the endoplasmic reticulum lumen, extracellular matrix, and cytoplasmic vesicle lumen in cellular components (Figure 3C). In addition, a comprehensive KEGG enrichment analysis was performed on the 63 common targets, revealing a total of 171 pathways (Figure 3D). These pathways mainly included the AGE-RAGE signaling pathway in diabetics, the lipid and atherosclerosis signaling pathway, the estrogen signaling pathway, and the neurotrophin signaling pathway. An effective compound–target–pathway network map was created using Cytoscape software (Figure 4). The bioactive compounds of CRP in this network are represented in yellow V, targets in red ellipses, and pathways in purple triangles. By utilizing this network, we found that each target was not isolated, and they could be affected by different compounds or act on different pathways. Therefore, this indicates that the interactions of multiple compounds, multiple targets, and multiple pathways play a significant role in the process of CRP ameliorating T2DOP.

### 3.6. Molecular Docking

**Figure 5:**
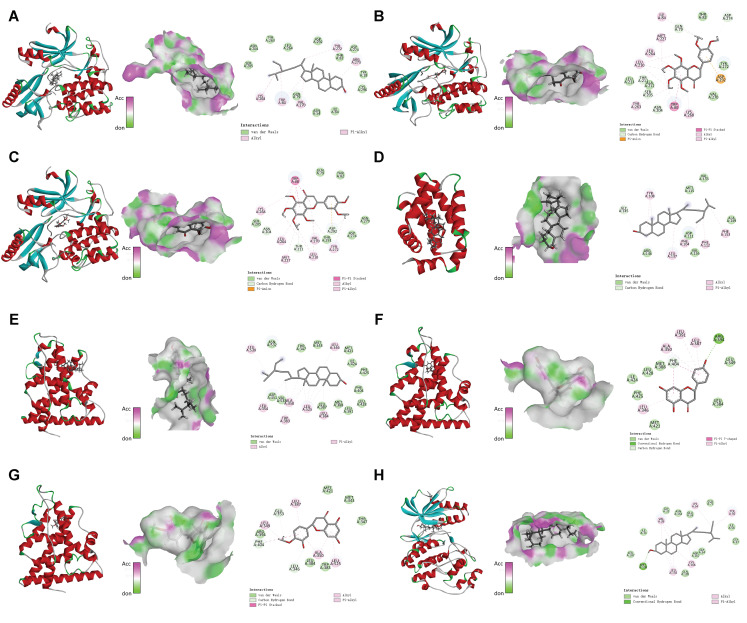
The eight sets of docking maps with the best molecular docking results. (**A**) AKT1-MOL000359; (**B**) AKT1-MOL005815; (**C**) AKT1-MOL005828; (**D**) BCL2-MOL000359; (**E**) ESR1-MOL000359; (**F**) ESR1-MOL004328; (**G**) ESR1-MOL005100; (**H**) MAPK1-MOL000359.

Citromitin, nobiletin, sitosterol, naringenin, and 5,7-dihydroxy-2-(3-hydroxy-4-methoxyphenyl), all of which are bioactive compounds found in CRP, were chosen for docking with the following core targets: AKT 1, TP 53, JUN, BCL 2, MAPK 1, NFKB 1, and ESR 1. A binding energy of <−5 kcal/mol generally indicates a significant capacity for spontaneous binding between the compound and the protein, whereas a binding energy of <−7 kcal/mol indicates a very stable binding between the compound and the protein. All of these compounds spontaneously bind to the core target, with binding energies from −5.1 to −11.2 kcal/mol, as revealed by the results (Table 2). However, the three proteins, AKT 1, MAPK 1, and ESR 1, and the five compounds showed more stable binding performances compared to the other groups (Figure 5A–H). Accordingly, the molecular docking results suggest that Citri Reticulatae Pericarpium is more likely to improve Type 2 diabetic osteoporosis through these pathways.

### 3.7. Molecular Dynamics Simulation

**Figure 6:**
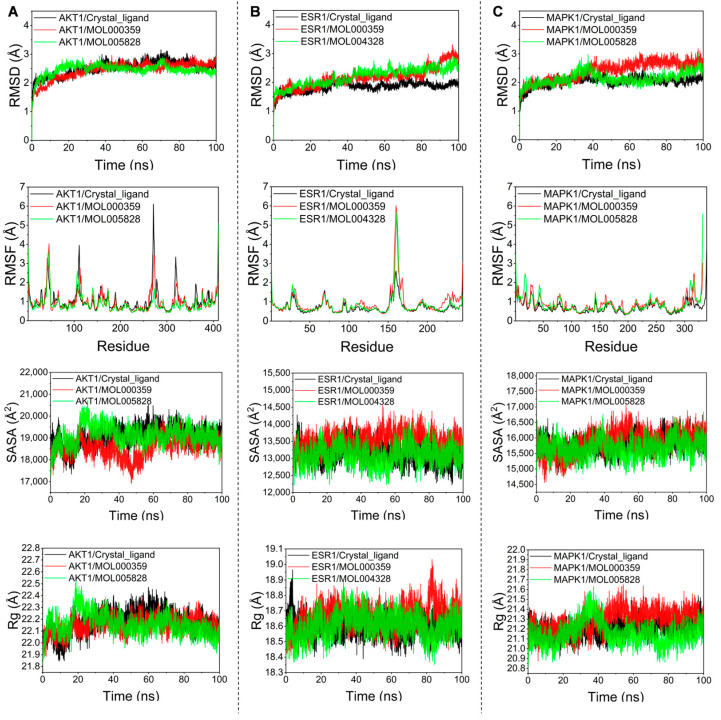
MD simulation of target proteins with bioactive compounds. (**A**) AKT1; (**B**) ESR1; (**C**) MAPK1.

Based on the above studies, we selected the three original proteins of AKT1, ESR1, and MAPK1 and the two best-performing bioactive compounds obtained from their docking with the molecule for molecular dynamics simulations. Root mean square deviation (RMSD) was used to measure the stability of proteins and protein–ligand complexes. It is generally believed that a protein’s stability increases, and its amplitude of change decreases, as the RMSD value decreases. As shown in Figure 6A–C, the maximal amplitude of the RMSD values for these bioactive compounds did not surpass 3.5 A, and they all fluctuated below 3.0 A. In contrast, throughout the simulations, the initial structures of all the complexes remained remarkably unchanged, suggesting that a system composed of these bioactive compounds and proteins would be highly stable. Root mean square fluctuation (RMSF) indicated the change in protein flexibility, and the results indicated that the reference molecule had a comparable effect on protein flexibility. Furthermore, solvent-accessible surface area (SASA) represents the stability of small molecules with protein atoms and changes in the exposed surface and buried regions of the complex during the simulation. Our results show that these complexes performed relatively well in SASA, with fluctuations and amplitudes similar or even superior to the original proteins. The radius of gyration (Rg) can be used to assess the tightness of small molecules and proteins, and the more stable the Rg value, the tighter the binding of the complex. In this study, the Rg values of the complexes with the original protein remained consistent with the contents of the other indicator responses.

### 3.8. Binding Free Energy Calculations of MMGBSA

The binding energy was determined by employing MMGBSA (molecular mechanics-generalized Born surface area) based on the trajectory of the molecular dynamics simulation. This approach enhanced the precision of the binding effects between reactive small molecules and target proteins. The final results show that the binding energy of these complexes ranged between −31 to −62.11 kcal/mol (Table 3, Table 4 and Table 5), with lower values indicating stronger binding and negative values indicating binding affinity between the small molecule and the target protein. It is evident that all of these active components bound to the core protein spontaneously and effectively. Moreover, energy decomposition revealed that the van der Waals energy exerted the greatest influence on the binding process in comparison to the nonpolar, solvation-free energy and electrostatic energy.

## 4. Discussion

Type 2 diabetic osteoporosis (T2DOP), as a systemic bone disease, has severe consequences on patients’ physical and mental well-being [38]. T2DOP is frequently brought on by a multitude of factors and is characterized by a complex pathogenesis and a wide range of symptoms. However, recent research has indicated that an efficacious treatment for this condition has yet to be identified [39]. Due to the variety of targets it addresses, traditional Chinese medicine (TCM) has become an increasingly popular area of study in recent years regarding the treatment of diseases with complex pathogeneses. This study aimed to identify the bioactive compounds present in Citri Reticulatae Pericarpium (CRP) that may have therapeutic potential for T2DOP. Additionally, the study investigated the primary targets and potential pathways of these bioactive compounds. As is complementary to this, the binding of the bioactive compounds and the core target was further validated by the molecular docking and molecular dynamics simulations.

Initially, we identified 63 common effect targets of the five bioactive compounds in CRP that are associated with T2DOP; these targets may be crucial for the treatment of T2DOP. Biological functions do not occur autonomously by a single protein, but rather by a network of proteins that is complex in nature. Seven core targets were identified through protein interaction network analysis of 63 common targets: AKT 1, TP 53, JUN, BCL 2, MAPK 1, NFKB 1, and ESR 1, respectively. These are the proteins in this protein network that interact more closely; thus, they might play a significant role in biological processes [40]. To validate this finding, we used molecular docking techniques to examine the five bioactive compounds of CRP separately from the core targets. The prospective relationship between the bioactive compounds of CRP and the core targets was supported by the results of molecular docking. Additionally, we found that AKT1, MAPK1, and ESR1 have better binding energy to the bioactive compounds. AKT1, a serine protein, is extensively expressed in various tissues and is primarily involved in regulating cellular proliferation and apoptosis. Studies have suggested that AKT1 might also be involved in the regulation of osteogenic and osteoclast differentiation [41,42]. MAPK1 is a vital protein for all organisms. It is involved in almost all the physiological processes of cells, as well as in the regulation of osteoclast differentiation, and affects bone health [43]. The crucial function of the MAPK signaling pathway in governing osteoblast differentiation and maturation has been demonstrated in many studies [44]. ESR1 is important for maintaining normal bone mass and bone metabolism, and is more significant for women. It also regulates the effects on the bones through multiple pathways, and in the ESR 1-Keap 1-Nrf 2 axis, ESR1 can interact with Nrf2 to promote osteogenic differentiation [45]; it can also affect the Wnt/β-catenin pathway and thus exert its influence on bone mineralization [46]. Moreover, there is evidence that several other targets also play important roles in osteogenic differentiation [47,48,49,50,51]. Thus, some theoretical basis can be established for the utilization of these core targets in the treatment of T2DOP.

The findings from the GO functional enrichment analysis indicated that Citri Reticulatae Pericarpium’s regulation of the oxidative stress response and hormone control could potentially serve as the primary therapeutic targets for type 2 diabetic osteoporosis. An increasing number of studies have suggested that oxidative stress plays a crucial role in the development and progression of osteoporosis. Elevated levels of oxidative stress inhibit the maturation of new osteoblasts and can accelerate osteoblast apoptosis [52]. An important feature of patients with osteoporosis is a long-term state of oxidative stress [53]. Furthermore, hormonal regulation, particularly estrogen regulation, exerts a significant influence on the progression of osteoporosis. This is due to its capacity to regulate bone metabolism and impact bone absorption and formation; it also has a protective effect on sclerotin [54]. Based on the findings from KEGG, CRP and its bioactive compounds exhibit greater enrichment in the AGE-RAGE signaling pathway, estrogen signaling pathway, and TNF signaling pathway, with the exception of the universal pathway. These pathways have been involved in the regulation of osteoporosis, according to the available evidence. Diabetic complications involve the AGE-RAGE signaling pathway, which is implicated in the development of diabetic bone disease. Hyperglycemia-associated advanced glycation end products (AGEs) have the potential to disrupt the regular processes of osteoblast and osteoclast development and differentiation, subsequently influencing the progression of osteoporosis [55]. At present, elevated concentrations of AGEs are considered a significant indicator of bone disorders [56]. Estrogen serves as the primary hormone modulator of bone metabolism, facilitating bone formation, enhancing bone density, and exerting a beneficial regulatory influence on diabetes and osteoporosis. Conversely, an insufficiency of estrogen prompts increased bone resorption, which ultimately disrupts the equilibrium of bone reconstruction. The occurrence of an inflammatory response is primarily attributed to TNF, which can also activate the NF-κB pathway [57]. NF-κB, being a transcription factor, plays a significant role in bone remodeling and metabolism. Moreover, through the construction of the active component–target–pathway network map, we found that these signaling pathways significantly enriched the core targets of the protein interaction network. These findings provide additional evidence that the treatment of T2DOP with CRP thoroughly exploits the combined effect of multiple bioactive compounds, targets, and pathways.

Studies have suggested that natural active substances possess distinct advantages when it comes to disease treatment. Moreover, the application of active substances in the treatment of bone diseases signifies important potential for advancement. In this study, the molecular docking results and other data indicated that the bioactive compounds sitosterol, naringenin, and nobiletin occupy a more important role in the treatment of type 2 diabetic osteoporosis with Citri Reticulatae Pericarpium. This relationship was further validated by molecular dynamics simulations, showing that the binding of these bioactive compounds to the core protein is comparable to, if not more effective than, that of the original ligand [58]. This could be further evidence of the mechanism and target by which CRP has the potential to treat T2DOP.

We acknowledge the limitations of the present investigation. Specifically, the targets and mechanisms of Citri Reticulatae Pericarpium and its bioactive compounds for the treatment of type 2 diabetic osteoporosis, obtained by using network pharmacology and molecular dynamics, are only reasonable predictions under the available data. Therefore, the authenticity of these predicted results still needs to be further verified by clinical studies and experiments. However, this study still has important research value, providing a new potential drug for the treatment of type 2 diabetic osteoporosis and revealing the potential mechanism of Citri Reticulatae Pericarpium for the treatment of type 2 diabetic osteoporosis.

## 5. Conclusions

Network pharmacology, molecular docking, and molecular dynamics simulation were utilized to investigate the underlying mechanism of Citri Reticulatae Pericarpium (CRP) and its bioactive compounds as a potential treatment for type 2 diabetic osteoporosis (T2DOP). Multiple bioactive compounds, targets, and pathways indicate that CRP may have a role in the treatment of type 2 diabetic osteoporosis. Furthermore, the binding performance of the bioactive compounds and core targets was assessed via molecular docking and molecular dynamics simulations, which provided additional support for the results shown above. Regarding the clinical application of CRP for T2DOP, this study serves as a guide and reference. The potential therapeutic application of CRP for the treatment of T2DOP requires further clinical and experimental investigations.

## Figures and Tables

**Table 1:** Bioactive components of Citri Reticulatae Pericarpium and their pharmacokinetic properties.

Mol ID	Molecule Name	OB (%)	DL
MOL000359	sitosterol	36.91	0.75
MOL004328	naringenin	59.29	0.21
MOL005100	5,7-dihydroxy-2-(3-hydroxy-4-methoxyphenyl)chroman-4-one	47.74	0.27
MOL005815	Citromitin	86.9	0.51
MOL005828	nobiletin	61.67	0.52

**Table 2:** Binding energy table of the bioactive compounds to the core targets.

PDB ID	Core Gene	MOL000359	MOL004328	MOL005100	MOL005815	MOL005828
5T01	JUN	−6.7	−5.7	−5.1	−5.4	−5.4
6S9W	AKT1	−11.2	−8	−7.3	−8.6	−8.6
7lhb	BCL2	−8.2	−7.3	−6.7	−6.5	−6.7
2YJA	ESR1	−8.4	−8.4	−8.2	−7.4	−7.2
6SLG	MAPK1	−8.7	−7.4	−6.7	−7.3	−7.6
3GUT	NFKB1	−6.7	−6.2	−6	−6	−6.1
7DHZ	TP53	−6.6	−7	−6.9	−5.9	−5.9

**Table 3:** Binding free energies and energy components of AKT1 predicted by MM/GBSA.

System Name	AKT1/Crystal_Ligand	AKT1/MOL000359	AKT1/MOL005828
ΔE_vdw_	−68.07 ± 1.56	−63.71 ± 1.82	−52.51 ± 2.05
ΔE_elec_	−27.54 ± 5.60	−2.20 ± 2.46	−13.59 ± 3.53
ΔG_GB_	67.76 ± 4.99	26.24 ± 1.79	41.47 ± 4.22
ΔG_SA_	−8.88 ± 0.32	−7.50 ± 0.06	−6.42 ± 0.15
ΔG_bind_	−36.74 ± 1.68	−47.18 ± 2.18	−31.05 ± 1.84

**Table 4:** Binding free energies and energy components of ESR1 predicted by MM/GBSA.

System Name	ESR1/Crystal_Ligand	ESR1/MOL000359	ESR1/MOL004328
ΔE_vdw_	−43.91 ± 2.20	−65.50 ± 1.08	−35.34 ± 1.51
ΔE_elec_	−8.07 ± 1.22	−1.66 ± 0.90	−24.76 ± 2.63
ΔG_GB_	18.13 ± 1.23	13.72 ± 0.88	32.16 ± 1.78
ΔG_SA_	−5.34 ± 0.10	−7.72 ± 0.05	−5.37 ± 0.10
ΔG_bind_	−39.19 ± 1.54	−61.16 ± 0.95	−33.31 ± 0.87

**Table 5:** Binding free energies and energy components of MAPK1 predicted by MM/GBSA.

System Name	MAPK1/Crystal_Ligand	MAPK1/MOL000359	MAPK1/MOL005828
ΔE_vdw_	−57.31± 2.84	−57.09 ± 2.56	−50.19 ± 2.73
ΔE_elec_	−48.87 ± 10.21	1.65 ± 1.30	−11.26 ± 3.86
ΔG_GB_	70.44± 9.18	27.54 ± 2.73	39.15 ± 2.44
ΔG_SA_	−7.25± 0.23	−6.73 ± 0.21	−6.65 ± 0.26
ΔG_bind_	−43.00± 2.11	−34.62 ± 1.37	−28.95 ± 2.05

## Data Availability

The data generated and/or analyzed in this study may be obtained from the corresponding author upon reasonable request.
